# Differential regulation of cell proliferation and protease secretion by epidermal growth factor and amphiregulin in tumoral *versus* normal breast epithelial cells

**DOI:** 10.1054/bjoc.2000.1678

**Published:** 2001-04

**Authors:** M Silvy, C Giusti, P-M Martin, Y Berthois

**Affiliations:** Laboratoire de Cancérologie Expérimentale, EA 2671, IFR Jean Roche, Faculté de Médecine secteur Nord, Bd Pierre Dramard, 13916 Marseille Cedex 20, France

**Keywords:** amphiregulin, epidermal growth factor, proteases, breast cancer cells

## Abstract

Amphiregulin (AR) is a heparin-binding epidermal growth factor (EGF)-related peptide that seems to play an important role in mammary epithelial cell growth regulation. We have investigated the regulation of AR-gene expression and -protein secretion by EGF in normal breast epithelial cells (HMECs), as well as in the tumoral breast epithelial cell lines MCF-7 and MDA-MB231. EGF induced a dose-dependent increase of AR mRNA level in both normal and tumoral cells. Thus, 10^−8^M EGF stimulated AR expression in HMECs to 140–300% of control. A similar EGF concentration increased AR mRNA level to 550% and 980% of control in MCF-7 and MDA-MB231 cells, respectively. This was accompanied by an accumulation of AR into conditioned culture media. However, HMECs secreted in response to EGF, 5–10 fold more AR than tumour cells. Furthermore, the potential participation of AR in the regulation of the plasminogen activator (PA)/plasmin system was investigated. Whereas HMEC-proliferation was stimulated by AR, the levels of secreted urokinase-type plasminogen activator (uPA) and type-1 plasminogen activator inhibitor (PAi-1) remained unaffected. Conversely, AR failed to regulate the proliferation of tumoral cell lines but induced an accumulation of uPA and PAi-1 into culture media. This was accompanied by an increase of the number of tumoral cells that invaded matrigel in vitro. Moreover, the presence of a neutralizing anti-uPA receptor antibody reversed the increased invasiveness of MDA-MB231 cells induced by AR. These data reveal differential behaviour of normal *versus* tumoral breast epithelial cells in regard to the action of AR and demonstrate that, in a number of cases, AR might play a significant role in tumour progression through the regulation of the PA/plasmin system. © 2001 Cancer Research Campaign http://www.bjcancer.com


*British Journal of Cancer* (2001) **84**(7), 936–945
© 2001 Cancer Research Campaign
doi: 10.1054/ bjoc.2001.1678, available online at http://www.idealibrary.com on IDEAL®

http://www.bjcancer.com

### Differential regulation of cell proliferation and protease secretion by epidermal growth factor and amphiregulin in tumoral *versus* normal breast epithelial cells

**M Silvy, C Giusti, P-M Martin and Y Berthois**

Laboratoire de Cancérologie Expérimentale, EA 2671, IFR Jean Roche, Faculté de Médecine secteur Nord, Bd Pierre Dramard, 13916 Marseille Cedex 20, France

**Summary** Amphiregulin (AR) is a heparin-binding epidermal growth factor (EGF)-related peptide that seems to play an important role in mammary epithelial cell growth regulation. We have investigated the regulation of AR-gene expression and -protein secretion by EGF in normal breast epithelial cells (HMECs), as well as in the tumoral breast epithelial cell lines MCF-7 and MDA-MB231. EGF induced a dose-dependent increase of AR mRNA level in both normal and tumoral cells. Thus, $10^{-8}$ M EGF stimulated AR expression in HMECs to 140–300% of control. A similar EGF concentration increased AR mRNA level to 550% and 980% of control in MCF-7 and MDA-MB231 cells, respectively. This was accompanied by an accumulation of AR into conditioned culture media. However, HMECs secreted in response to EGF, 5–10 fold more AR than tumour cells. Furthermore, the potential participation of AR in the regulation of the plasminogen activator (PA)/plasmin system was investigated. Whereas HMEC-proliferation was stimulated by AR, the levels of secreted urokinase-type plasminogen activator (uPA) and type-1 plasminogen activator inhibitor (PAi-1) remained unaffected. Conversely, AR failed to regulate the proliferation of tumoral cell lines but induced an accumulation of uPA and PAi-1 into culture media. This was accompanied by an increase of the number of tumoral cells that invaded matrigel in vitro. Moreover, the presence of a neutralizing anti-uPA receptor antibody reversed the increased invasiveness of MDA-MB231 cells induced by AR. These data reveal differential behaviour of normal *versus* tumoral breast epithelial cells in regard to the action of AR and demonstrate that, in a number of cases, AR might play a significant role in tumour progression through the regulation of the PA/plasmin system. © 2001 Cancer Research Campaign http://www.bjcancer.com

**Keywords**: amphiregulin; epidermal growth factor; proteases; breast cancer cells

Numerous well-characterized growth factors play an important role in regulating the growth and development of the mammalian mammary gland. The expression and secretion of some of these growth factors are under the control of ovarian hormones and probably act as important autocrine and paracrine modulators that can regulate end bud, ductal and alveolar growth and development. Likewise, a number of growth factors and growth inhibitors, including EGF, TGFα TGFβ, amphiregulin, Cripto-1, heregulin, heparin binding-EGF and plateled-derived growth factor are synthesized by malignant mammary epithelial cells (Ciardiello et al, 1989; Normanno et al, 1994a, b; Qi et al, 1994). Most of them have been demonstrated to function as either positive or negative autocrine growth regulators in vitro in a variety of human breast cancer cell lines (Kenney et al, 1993; Souttou et al, 1994).

A number of growth factors are also described to participate in the regulation of the plasminogen activator (PA)/plasmin system (Rosenthal et al, 1998). PA are serine proteases, catalysing the conversion of plasminogen to plasmin. Plasmin exhibits a wide array of physiological and pathological processes, such as tissue growth and remodelling, tumour invasion and metastasis (Andreasen et al, 1997). Two classes of proteases, serine proteinases and the metalloproteinases, and their inhibitors, have been studied extensively in breast and other cancers. Many studies have focused on the role of urokinase-type plasminogen activator (uPA) and have described a correlation between uPA expression and cell invasion and metastasis (Foeckens et al, 1992). Type-1 plasminogen activator inhibitor (PAi-1) may also be directly involved in cancer progression, and an increasing number of clinical studies have demonstrated that high PAi-1 levels indicate a poor prognosis for the survival of patients suffering from a variety of cancers (Bouchet et al, 1994).

Among EGF-related growth factors, amphiregulin (AR) seems to play special physio-pathological role in the human mammary gland. Amphiregulin is a heparin-binding growth factor initially purified from conditioned medium of breast cancer epithelial MCF-7 cells treated with phorbol 12-myristate 13-acetate (Shoyab et al, 1988). Whereas AR binds and activates EGF-receptor (c-erb B1), direct interaction of AR with other members of the c-erb B family has not been reported (Johnson et al, 1993a). AR is expressed in several tissues, such as human ovary, placenta, lung, kidney, stomach, colon and breast (Johnson et al, 1992; Lejeune et al, 1993). Increased evidences strongly suggest that AR may function as a potential autocrine growth factor in tumoral as well as in their counterpart normal cells, such as prostatic (Sehgal et al, 1994), colonic (Johnson et al, 1992; Normanno et al, 1995), and mammary epithelial cells (Li et al, 1992; Normanno et al, 1994a, b). Moreover, overexpression of AR has been detected in several oestrogen-responsive and -unresponsive breast cancer cell lines as well as in approximately 80% of human primary breast carcinoma (Martinez-Lacaci et al, 1995; Visscher et al, 1997). More important, overexpression of AR, but not that of TGFα or Cripto, appears as an independent prognostic indicator correlated with a diminution of survival for patients with non-small-cell lung cancer (Fontanini et al, 1998).

*Received 28 July 2000*
*Revised 3 January 2001*
*Accepted 4 January 2001*

*Correspondence to: Y Berthois*

Whereas the expression of AR in a variety of both non-transformed and tumoral cells appears to be stimulated in the presence of EGF (Normanno et al, 1994b; Sehgal et al, 1994), the potential regulation of AR by growth factors in normal breast epithelial cells has never been examined. Because previous studies suggest that dysregulated expression of AR may be a component of mammary tumorigenesis we proposed to analyse and to compare the regulation of amphiregulin gene expression and protein secretion by EGF in normal and tumoral breast epithelial cells. Moreover, our aim was to examine potential role of AR in the regulation of uPA and PAi-1 protein secretion that could account for breast cancer progression.

### MATERIAL AND METHODS

### Materials

Anti-smooth muscle α-actin, -actin, -vimentin and -cytokeratin 14, 18 and 19 antibodies were provided by Sigma (St Quentin Fallavier, France); Anti-vimentin antibody was from DAKO (Denmark). The AR cDNA probe was obtained from Dr. Plowman (Bristol-Myers Squibb, Seattle, WA). Matrigel was provided by Becton-Dickinson (France). Anti-uPA receptor neutralizing antibody, recombinant human AR and AR enzyme-immunoassay kit were from R & D Systems (Abingdon, UK). Antibodies used in ELISA determination of AR recognize various forms of promature and mature human AR of 12–46 kDa. They show no cross-reactivity with other members of the EGF-related growth factors, as well as with a large variety of other cytokines. PAi-1 and uPA ELISA kits were from American Diagnostic Inc (Greenwich, CT). PAi-1 ELISA detects latent and active forms of human PAi-1 and PAi-1 complexes. The assay is insensitive to PAi-2. Inactive and active forms of uPA are all recognized by the uPA ELISA kit, as is receptor-bound uPA and uPA complexed with PAi-1 and PAi-2.

### Culture of breast cancer epithelial cell lines

The MCF-7 cell line, derived from human breast adenocarcinoma was obtained from Dr M Lippman (NIH, Bethesda). Cells were maintained in 50% (vol/vol) Dulbecco's modified Eagles medium (DMEM) and 50% (vol/vol) Ham's F12 medium (F12), supplemented with 10% heat-inactivated fetal calf serum (FCS), 2 mM L-glutamine and 6 ng $ml^{-1}$ human insulin. The MDA-MB231 cells obtained from Mason Research Institute were cultured in Leibovitz L15 medium containing 10% FCS, 2 mM L-glutamine, 10 mM Hepes, 0.01% non essential amino acids (Gibco BRL) and 3.6 μg $ml^{-1}$ insulin. MCF-7 cells were maintained at 37˚C in a humid atmosphere of 5% $CO_2$ in air, and MDA-MB231 cells in a humid atmosphere of air. Before experimentation, cells were grown for one week in medium containing steroid-depleted serum.

### Primary culture of normal breast epithelial cells

Epithelial cell cultures were grown from tissue specimens obtained after their informed consent from 5 women between 16 and 44 years of age who underwent reduction mammoplasty for cosmetic reasons. The time of the menstrual cycle was noted for every patient. These patients had no history of breast disease and normal state of breast samples was verified by histo-pathological examination. Tissues were mechanically dissociated with scissors then incubated at 37˚C with constant shaking in medium containing 150 U $ml^{-1}$ hyaluronidase (Sigma) and 250–500 U $ml^{-1}$ collagenase (Sigma). Digestion was monitored under an inverted microscope. Released organoids were then separated by density gradient centrifugation using 1.077 g $ml^{-1}$ lymphocyte separation medium. Organoids were then plated in DMEM/F12 (1/1, vol/vol) containing 10 mM Hepes, 2 mM glutamine, 10 μg $ml^{-1}$ insulin, $5 \times 10^{-6}$ M cortisol, 50 U $ml^{-1}$ penicillin, 50 mg $ml^{-1}$ streptomycin, 100 ng $ml^{-1}$ cholera toxin, 2 ng $ml^{-1}$ EGF and 5% FCS ($B_1$ medium). Cells were cultured at 37˚C in a 5% $CO_2$–95% air atmosphere. One week following the plating, medium was replaced by the same medium containing 60 μM $CaCl_2$ instead of 1.05 mM ($B_2$ medium). Cells were sub-cultured by the transfer of floating cells. Before experimentation, cells were grown for one week in steroid-depleted $B_1$ medium deprived of EGF and cholera toxin. The epithelial phenotype of cultured cells was verified by immuno-cytochemistry, using monoclonal antibodies specific for the following proteins: cytokeratins 14, 18 and 19, smooth muscle α-actin, actin and vimentin.

### Cell proliferation assay

Cell growth determination was performed in 6 well-culture plates. Cells were seeded in culture medium containing 1% steroid-depleted serum. They were allowed to grow for 3 days then treated with different concentrations of EGF or AR ($10^{-12}$ M–$10^{-8}$ M). Treatments were performed for 5 days and renewed every 2 days. At the end of the treatment, cells were harvested and cell number was determined using a cell counter.

### Preparation of conditioned medium, and amphiregulin and protease measurements

Cells were seeded in 6 well-culture plates, in culture medium supplemented with 1% steroid-depleted FCS. 3 days later, cells received different concentrations of EGF or AR ($10^{-10}$ M–$10^{-8}$ M). Cells were treated for 2 days, then treatments were continued in serum-free culture medium for 24 h. Conditioned media were collected and particulates were removed by centrifugation. Quantitative determination of human amphiregulin, PAi-1 and uPA concentrations was performed using enzyme-immunoassay kits, according to the instructions of the manufacturers.

### Invasion assay

Invasion assays were carried out in Transwell culture system (Costar Corporation, France). Briefly, 6.4 mm-diameter filters (8 μm pores) of cell culture inserts were coated with 12 μg $filter^{-1}$ of reconstituted basement membrane Matrigel diluted with ice-cold distilled water. Matrigel was allowed to dry overnight then reconstituted with DMEM/F12 medium for 90 min at room temperature. Uniformity of the coating was verified by coomassie blue staining and microscopic observation. Exponentially growing cells were plated in the inserts (15 000 cells $insert^{-1}$). MCF-7 and MDA-MB231 cells were grown in their respective culture medium containing 1% steroid-depleted FBS. NBECs were cultured in EGF- and cholera toxin-free B1 medium supplemented with 1% steroid-depleted FBS. The inserts were then placed into the wells of 24 well-culture plates. Amphiregulin at $10^{-8}$ M was added in the upper compartment, whereas fibronectin (15 μg $ml^{-1}$) was placed in the lower compartment as a chemoattractant. Culture medium and treatments were renewed every 2 days. After

© 2001 Cancer Research Campaign 

incubation at 37˚C for 5 days, the cells on the upper surface of the filter were removed with a cotton swab. The cells that had traversed the matrigel and attached to the lower part of the filter were fixed, stained with Harris haematoxylin and counted in 15 randomly selected microscopic fields.

### Isolation of cellular RNA

Cells were plated into 150 $cm^2$ T-flasks in medium containing 1% steroid-depleted serum. 3 days later cells were treated in the absence or in the presence of various concentrations of EGF ($10^{-10}$ M–$10^{-8}$ M). Treatments were performed for 2 days, then RNA extraction was carried out using RNazol (Cinna-Biotex, Friendswood, Texas, USA). After addition of equal volumes of chloroform, extracted RNA was precipitated with one volume of isopropanol. After washing, pellet was resuspended in 20–40 µl DEPC-water.

### Reverse transcription

First strand cDNA was synthesized by using Moloney mouse leukaemia virus (MMLV) reverse transcriptase. 5 µg of total RNA extracted as previously described was incubated with oligo(dT) (1 mg per reaction) and DEPC-water (*qsp* 6 µl) for 10 min at 65˚C, then for 5 min at 0˚C. Reverse transcription was then initiated in the presence of 2 µl of MMLV (400 U), 5 µl of 5-fold concentrated reaction buffer (250 mM Tris-HCl pH 8.3, 375 mM KCl, 15 mM $MgCl_2$), 1 µl of 10 mM dNTP, 2 µl of 100 mM dithiothreitol and 0.5 µl RNase inhibitor (40 U $ml^{-1}$). Reaction was performed for 1 h at 37˚C then for 30 min at 52˚C.

### Polymerase chain reaction and Southern blot analysis

Co-amplifications of the AR and $\beta_2$-microglobulin cDNAs, and Southern blot analysis of the PCR products were performed as reported previously (Silvy et al, 1998). Briefly, 30 µl of the PCR products were separated by electrophoresis in 3% agarose gel. The gel was stained with ethidium bromide to allow the visualization of DNA that was then denatured and transferred to a Hybond N membrane (Amersham). The amphiregulin-cDNA probe was labelled with [$\alpha$-$^{32}$P]dCTP using the random priming method (Feinberg and Vogelstein, 1983). The $\beta_2$-microglobulin probe was labelled with [$\gamma$-$^{32}$P]dCTP using the T4 kinase enzyme.

### Image acquisition and analysis

The autoradiographies were scanned with the AGFA ARCUS TM scanner (Agfa-Geavaert AG) in the transparency mode. The resolution of the scanner is adjustable from 1 to 1200 pixel per inch (ppi). The software used in the analysis was the Macintosh-based public domain program Image, written by Rasband at the National Institute of Health (NIH). The program contains a built-in macro language permitting complicated repetitive procedures to be converted to single commands as we use here. The Integrated Optical Density (IOD) of each band was measured with a background subtraction.

### Statistical analysis

All values were expressed as mean ± SE. Student's *t*-test was used for comparisons between experimental data. A value of $P < 0.05$ was considered to be statistically slightly significant (*); a value of $P < 0.01$, statistically significant (**); and a value of $P < 0.001$, highly significant (***).

### RESULTS

### Immunophenotyping of normal human mammary epithelial cells

The epithelial phenotype of normal human mammary epithelial cells (HMECs) was verified by immuno-cytochemical assay (Table 1). The positive immuno-staining for cytokeratins 18 and 19 in the majority of the cells confirmed the epithelial nature of the primary cultures. In some primary cultures, very weak vimentin- and cytokeratin 14-stainings were observed in less than 20% of cells and the large majority of cells appeared negative for actin, suggesting the almost total absence of myoepithelial cells. Moreover, smooth muscle α-actin appeared to be undetectable in all the samples analysed.

### Effect of EGF on AR expression and secretion in normal and tumoral breast epithelial cells

The effect of EGF on AR expression was examined in the breast cancer cell lines MCF-7 and MDA-MB231 as well as in a panel of normal breast epithelial cells in primary culture. The variations

**Table 1** Immunophenotyping of normal breast epithelial cells in primary culture

| Sample no. | Age | CK14 | CK18 | CK19 | α-Actin | Actin | Vimentin |
|---|---|---|---|---|---|---|---|
| SN52 | 16 | + (<20) | ++ (>90) | + (>80) | – | – | + (<20) |
| SN53 | 26 | + (<20) | ++ (>90) | + (>90) | – | – | + (<20) |
| SN48 | 28 | – | ++ (>90) | ++ (>90) | – | – | – |
| SN47 | 44 | – | ++ (>90) | + (>80) | – | – | + (<20) |
| SN50 | ND | – | ++ (>90) | ++ (>90) | – | – | – |

The epithelial phenotype was verified by immunocytochemical assay as described in 'Materials and methods'. –, no staining; +, low staining; ++, strong staining. Numbers in parenthesis indicate the percentage of positive cells. ND, not determined.

© 2001 Cancer Research Campaign

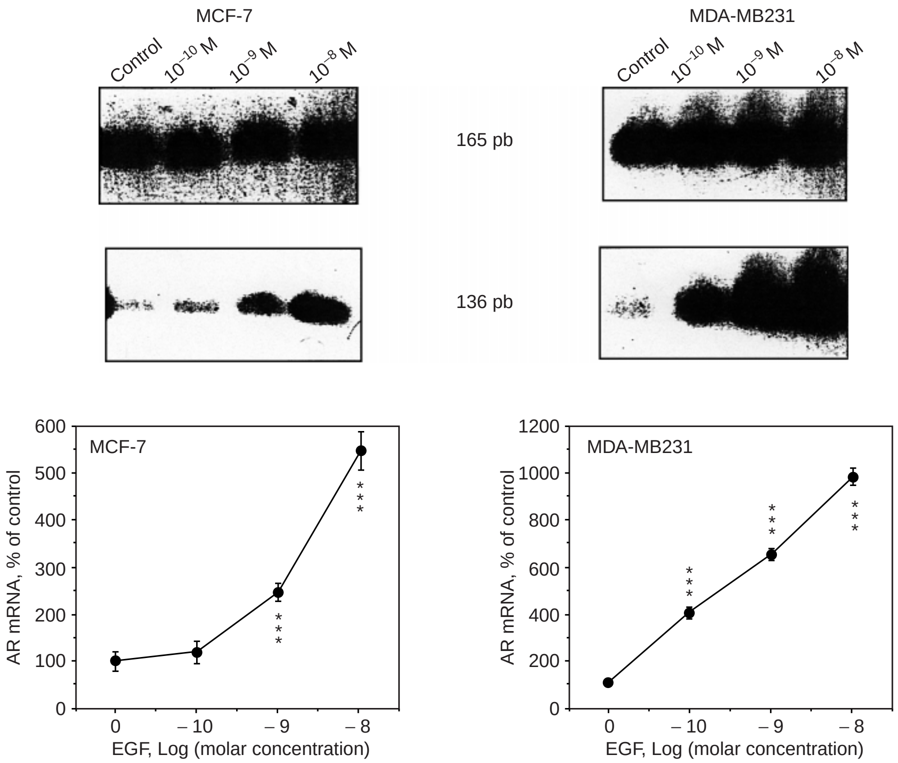

**Figure 1** Effect of EGF on AR mRNA level in MDA-MB231 and MCF-7 cells. Studies were performed on cells treated for 48 h with EGF at the indicated concentrations. AR and $\beta_2$-microglobulin mRNA levels were assayed by RT-PCR. The 165 pb $\beta_2$-microglobulin and 136 bp AR amplified fragments were probed as described in Material and Methods with [$\alpha$-$^{32}$P]dCTP-labelled probes. After autoradiography, the integrated optical density of each band was measured with a background subtraction as indicated in Material and Methods. Values obtained for each sample were corrected for the corresponding $\beta_2$-microglobulin mRNA level. Data are the mean ± SD of values obtained from 3 separate experiments and are expressed as percentage of control. ***, significantly different from the control by Student's test with $P < 0.001$

of AR mRNA level induced by various concentrations of EGF were evaluated by RT-PCR. In both tumoral cell lines, EGF was demonstrated to induce an important stimulation of AR expression (Figure 1). However, the augmentation of AR mRNA level induced by EGF was more elevated in MDA-MB231 than in MCF-7 cells. Thus, cell treatment with $10^{-8}$ M EGF increased AR mRNA level to 550% and 980% of control in MCF-7 and MDA-MB231 cells, respectively. A dose-dependent accumulation of the secreted protein in the culture medium of both cell lines was also observed in response to EGF. However, the amplitude of this accumulation was moderate (Table 2). Thus, in the presence of $10^{-8}$ M EGF, the level of AR secreted by MCF-7 cells attained 270% of control. In the MDA-MB231 cell line, a similar EGF concentration induced a slight but significant augmentation of secreted AR (180% of control). Decreased doses of EGF were unable to affect the accumulation of AR in culture medium of these cells. Moreover, the level of cell-associated AR appeared little affected by EGF since AR level measured in cell homogenate attained 130% and 150% of control in MDA-MB231 and MCF-7 cells, respectively (Table 2).

EGF was also able to increase AR expression in normal breast epithelial cells in primary culture. The EGF-induced AR mRNA stimulation was dose-dependent and varied from 140% to 350% of

**Table 2** Effect of EGF on the level of AR protein in tumoral breast epithelial cells

| | AR, pg per $10^6$ cells | | | |
|---|---|---|---|---|
| | Conditioned medium | | Cell homogenate | |
| | MCF-7 | MDA-MB231 | MCF-7 | MDA-MB231 |
| Control | 75 ± 8 (100) | 53 ± 11 (100) | 56 ± 12 (100) | 36 ± 10 (100) |
| EGF $10^{-10}$ M | 60 ± 7 (80) | 52 ± 9 (98) | 51 ± 5 (91) | 42 ± 4 (116) |
| EGF $10^{-9}$ M | 155 ± 21 (206) | 50 ± 4 (94) | 63 ± 13 (112) | 40 ± 10 (111) |
| EGF $10^{-8}$ M | 203 ± 19 (270) | 97 ± 11 (180) | 86 ± 12 (153) | 48 ± 11 (130) |

Cell homogenates and conditioned medium produced by untreated and EGF-treated cells was prepared as described in 'Material and Methods' and the level of AR was determined by enzyme-immunoassay. The data are the mean +/– SD of two separate experiments. Values in parenthesis are expressed as percentage of control.

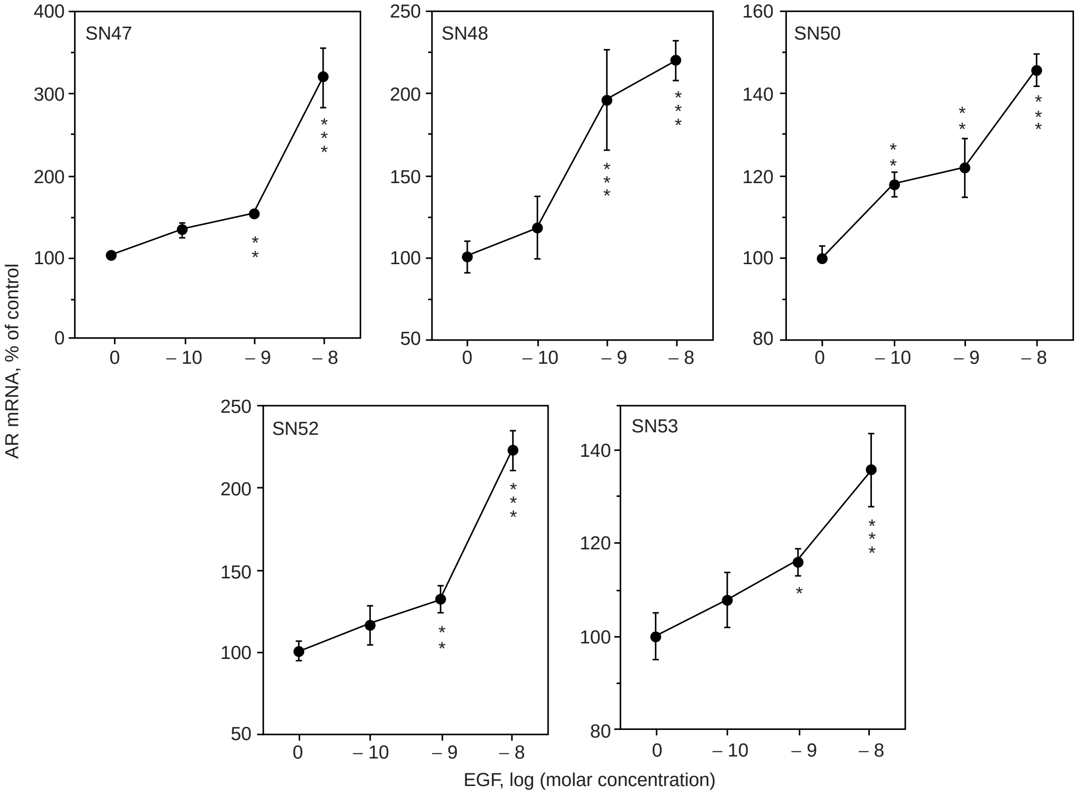

**Figure 2** Effect of EGF on AR mRNA level in normal mammary epithelial cells in primary culture. Studies were performed on 5 different samples, treated for 48 h with EGF at the indicated concentrations. AR and $\beta_2$-microglobulin mRNA expression levels were assayed by RT-PCR and Southern blot analysis as described in Materials and Methods. Values obtained for each sample were corrected for the corresponding $\beta_2$-microglobulin mRNA level. Data are the mean ±SD of values obtained from 3 separate experiments and are expressed as percentage of control. *, **, and ***, significantly different from the control by Student's test with $P < 0.05$, $P < 0.01$ and $P < 0.001$, respectively control, depending upon the samples analysed (Figure 2). This was accompanied by an important augmentation of the level of secreted protein. Thus, in the presence of $10^{-8}$ M EGF, the accumulation of AR in conditioned medium produced by the normal epithelial cells SN52 and SN47 attained 550% and 680% of control, respectively (Table 3). Similar high level of protein accumulation was observed for SN53, SN48 and SN50 cells when treated with EGF (not shown). By contrast, moderate increase (150–200%) of cell-associated AR could be detected in HMEC homogenates in response to EGF (Table 3).

### Effect of AR and EGF on cell proliferation and protease secretion in normal and tumoral breast epithelial cells

The ability of AR to regulate cell proliferation, PAi-1 and uPA secretion was evaluated in normal and tumoral breast epithelial cells. As illustrated in Figure 3, AR added in cell culture medium was unable to affect the proliferation of the breast cancer epithelial cell lines MCF-7 and MDA-MB231. On the contrary, AR induced a stimulation of PAi-1 and uPA secretion by MDA-MB231 cells, in a dose-dependent manner. Thus, treatment of these cells with

**Table 3** Effect of EGF on the level of AR protein in normal breast epithelial cells

| | AR, pg per $10^6$ cells | | | |
|---|---|---|---|---|
| | Conditioned medium | | Cell homogenate | |
| | SN52 | SN47 | SN52 | SN47 |
| Control | 204 ± 22 (100) | 413 ± 51 (100) | 180 ± 19 (100) | 216 ± 31 (100) |
| EGF $10^{-10}$ M | 462 ± 28 (226) | 500 ± 35 (120) | 172 ± 23 (95) | 241 ± 17 (111) |
| EGF $10^{-9}$ M | 626 ± 71 (306) | 1166 ± 53 (280) | 256 ± 21 (140) | 392 ± 32 (181) |
| EGF $10^{-8}$ M | 1125 ± 83 (551) | 2833 ± 112 (681) | 281 ± 29 (156) | 436 ± 26 (202) |

Cell homogenates and conditioned medium produced by untreated and EGF-treated cells was prepared as described in 'Material and Methods' and the level of AR was determined by enzyme-immunoassay. The data are the mean ± SD of two separate experiments. Values in parenthesis are expressed as percentage of control. Similar results were obtained for SN53, SN48 and SN50 normal breast epithelial cells.

*© 2001 Cancer Research Campaign*

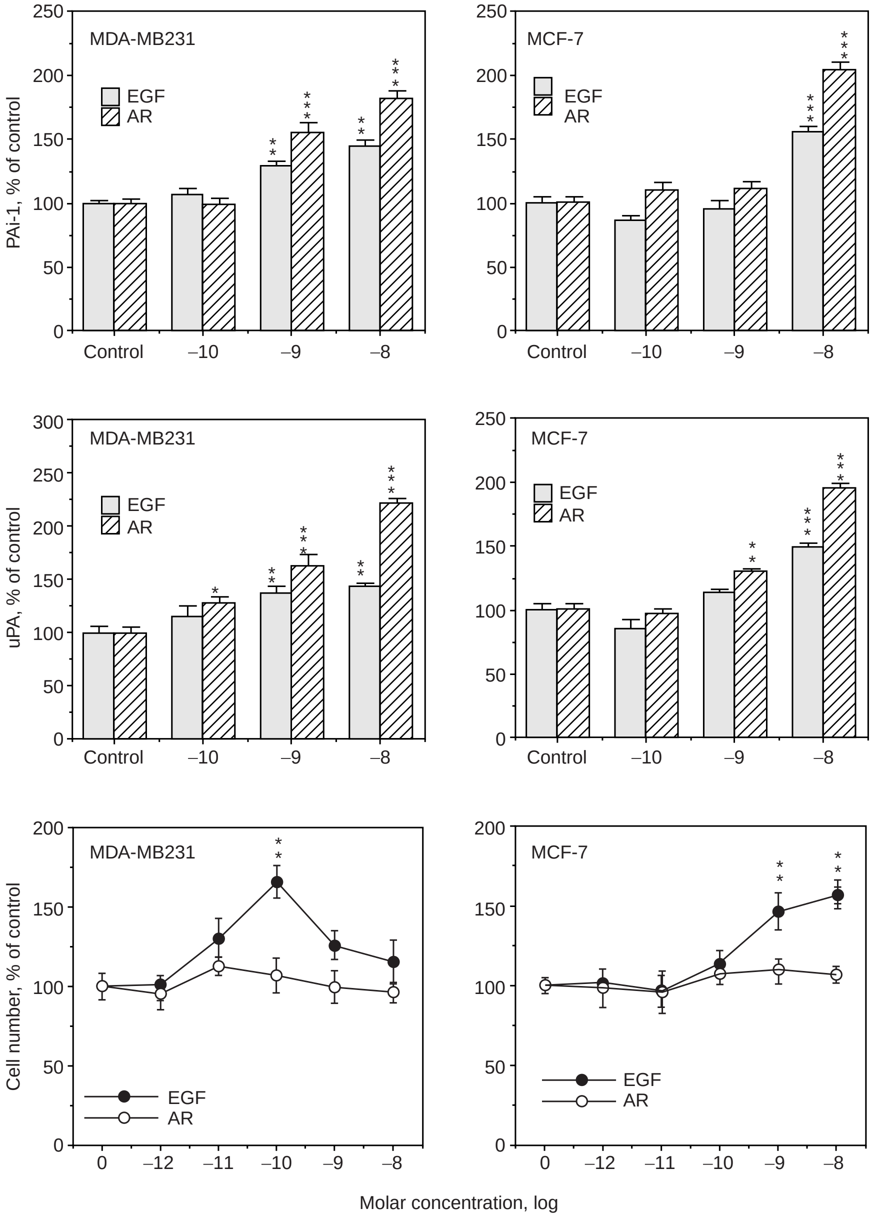

**Figure 3** Effect of EGF and AR on the proliferation, PAi-1 and uPA production by MDA-MB231 and MCF-7 cells. Cells were seeded in culture medium containing 1% steroid-depleted serum and treated for 2 days with various concentrations of AR or EGF. Cells were then placed and treated in serum-free culture medium for 24 h. Conditioned medium was collected and uPA and PAi-1 levels were determined by enzyme-immunoassay as described in Materials and Methods. Basal levels of PAi-1 and uPA for MDA-MB231 cells were 85.3 ± 3.4 ng mg$^{-1}$ protein and 23.3 ± 2.30 ng mg$^{-1}$ protein, respectively. For MCF-7 cells, PAI-1 and uPA basal levels were 356.2 ± 17.8 ng mg$^{-1}$ protein and 1.51 ± 0.06 ng mg$^{-1}$ protein, respectively. In parallel, the effect of EGF and AR on cell proliferation was assayed by the determination of cell number. All data are expressed as percentage of control and are the mean ± SD of values obtained from 3 separate experiments. *, **, and ***, significantly different from the control by Student's test with $P < 0.05$, $P < 0.01$ and $P < 0.001$, respectively

$10^{-8}$ M AR increased both PAi-1 and uPA accumulation in conditioned medium to 160% and 220% of control, respectively. Similarly, in the presence of $10^{-8}$ M AR, the levels of PAi-1 and uPA secreted by MCF-7 cells were augmented to 200% of control. In both tumoral cell lines, a slight augmentation (120–150% of control) of cell-associated uPA and PAi-1 levels was detected in response to $10^{-8}$ M AR (not shown). In parallel, the effect of EGF on cell proliferation and protease secretion was also examined. A moderate stimulation (160% of control) of MDA-MB231 cell proliferation was induced by $10^{-10}$ M EGF. A dose-dependent growth stimulation of MCF-7 cell line that attained 150% of control in the presence of $10^{-8}$ M EGF was observed. EGF was also able to induce an accumulation of uPA and PAi-1 into conditioned medium of both tumour cell lines. However, it appeared less efficient than AR in stimulating protease production.

In order to compare the biological activity of AR in normal *versus* tumoral mammary tissue, a similar study was carried out on a panel of normal mammary breast epithelial cells (HMECs) in primary culture. Data reported in Figure 4 indicate that AR was able to induce a dramatic dose-dependent stimulation of normal epithelial cell proliferation. Thus, a growth-stimulatory effect was observable by $10^{-10}$ M AR. In the presence of $10^{-8}$ M AR, HMEC

© 2001 Cancer Research Campaign

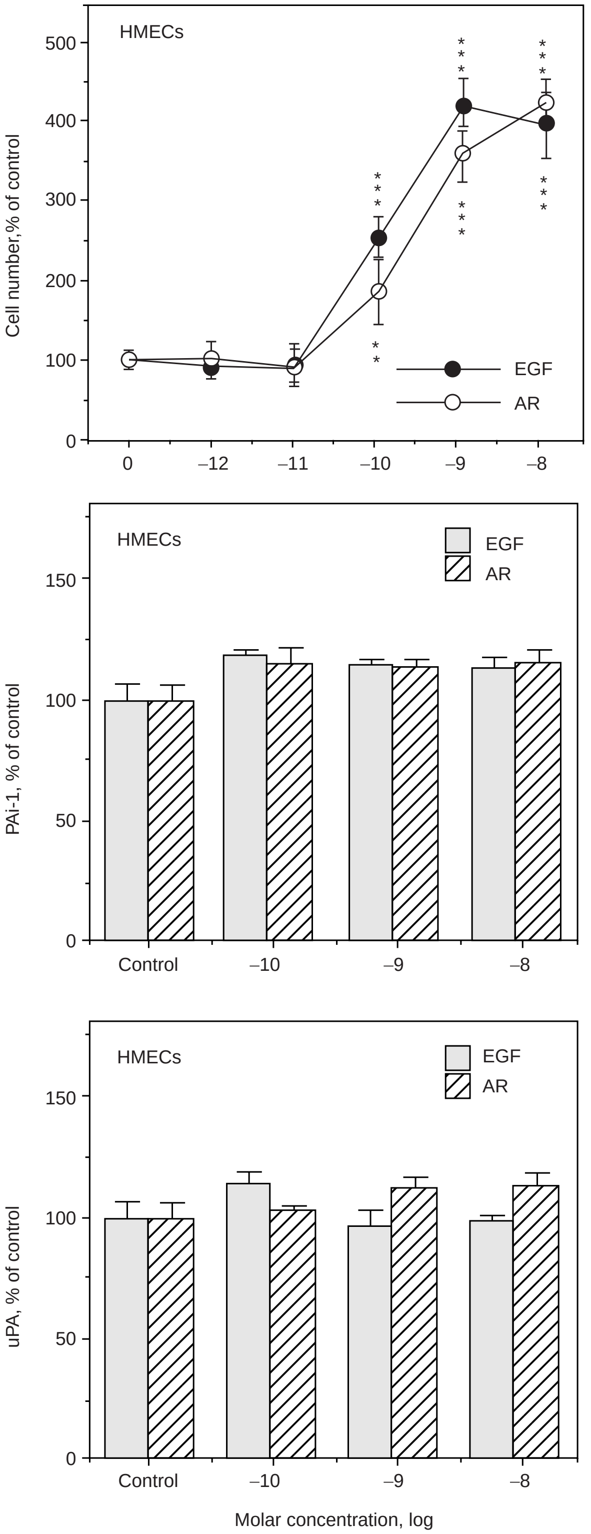

**Figure 4** Effect of EGF and AR on the proliferation, PAi-1 and uPA production by normal mammary epithelial cells in primary culture (HMEC). Experiments were performed as described in Figure 3. The results presented have been obtained with HMECs SN48. Basal levels of PAi-1 and uPA measured into conditioned culture medium were 318.7 ± 29.6 ng mg$^{-1}$ protein and 8.63 ± 0.95 ng mg$^{-1}$ protein, respectively. The samples SN47 and SN50 were also examined and allowed to similar results. Data are the mean ± SD of triplicate determinations and are representative of 3 separate experiments. All data are expressed as percentage of control. **, and ***, significantly different from the control by Student's test with $P < 0.01$ and $P < 0.001$, respectively

**Table 4** Effect of AR and anti-uPA-R antibody on the invasiveness of MCF-7 and MDA-MB231 cells

| | Number of invasive cells (% of control) | |
|---|---|---|
| **Treatments** | **MDA-MB231** | **MCF-7** |
| Control | 100 ± 6.2 | 100 ± 5.4 |
| AR $10^{-10}$ M | 140 ± 6.0[a] | 107 ± 8.3 |
| AR $10^{-9}$ M | 197 ± 3.3[b] | 143 ± 5.7[a] |
| AR $10^{-8}$ M | 220 ± 8.3[b] | 162 ± 2.9[b] |
| α uPA–R | 64 ± 2.4 | 97 ± 6.6 |
| AR $10^{-8}$ M +αuPA-R | 112 ± 5.6[c] | 166 ± 3.9 |

Exponentially growing cells were harvested and added to invasion chambers in the absence or in the presence of different concentrations of AR. The involvement of uPA in cell invasiveness was evaluated by adding 10 μg ml$^{-1}$ anti-uPA-R antibody (α uPA-R), in the absence or in the presence of $10^{-8}$ M AR. Data are the mean ± SD of 3 independent experiments and are expressed as the percentage of control (untreated cells). [a], $P < 0.01$ versus control; [b], $P < 0.001$ versus control; [c], $P < 0.001$ versus $10^{-8}$ M AR.

proliferation was stimulated to 400–500% of control, depending on the samples examined. On the contrary, the accumulation of both uPA and PAi-1 in conditioned medium of HMECs was not affected by AR. In parallel, it was verified that the levels of cell-associated uPA and PAi-1 were not modified in the presence of AR (not shown). Similar results, regarding cell proliferation and protease secretion, were obtained with EGF.

### Effect of AR on the invasive properties of MCF-7 and MDA-MB231 cells

The addition of $10^{-10}$ M–$10^{-8}$ M AR in the upper compartment of the invasion chamber allowed to a significant dose-dependent increase of the number of tumoral cells that invaded the Matrigel membrane (Table 4). Time course analysis indicated that the number of invasive cells was maximal after 4–5 days of culture (not shown). However, MDA-MB231 cells appeared more responsive than MCF-7 cells to the invasive effect of AR. Thus, the presence of $10^{-8}$ M AR augmented the number of MCF-7 and MDA-MB231 cells that had traversed the basement membrane to 160% and 220% of control, respectively. Moreover, whereas $10^{-9}$ M AR was required to observe a significant increase of the number of invasive MCF-7 cells (143% of control), a concentration of $10^{-10}$ M AR appeared sufficient to significantly stimulate the invasive properties of MDA-MB231 cells. Conversely, untreated HMECs appeared unable to traverse matrigel, suggesting that, under our experimental conditions, uPA may be produced as latent (inactive) forms. Furthermore, the presence of AR ($10^{-10}$ M–$10^{-8}$ M) could not induce significant migration of HMECs, even after 5 days of culture (not shown).

In order to further examine the potential role of uPA in the increased invasiveness of tumoral cells treated with AR, a neutralizing anti-uPA receptor antibody was added into culture medium, in the absence or in the presence of $10^{-8}$ M AR. Results reported in Table 3 indicate that the presence of the antibody was unable to affect the invasive properties of both AR-treated and -untreated MCF-7 cells. On the contrary, a moderate reduction of the number of MDA-MB231 cells able to invade matrigel was observed when cells were treated with the antibody alone. Furthermore, the antibody totally reversed the stimulatory effect of $10^{-8}$ M AR on the invasive activity of these cells, demonstrating that the increased

© 2001 Cancer Research Campaign invasiveness of MDA-MB231 cells induced by AR was mediated in large part by uPA.

### DISCUSSION

AR, which has been described as a potent stimulator of proliferation in a variety of cell types, is expressed by epithelial cells in a number of normal human tissues including the mammary gland (Li et al, 1992; Panico et al, 1996). Moreover, AR has been shown to act as an autocrine growth factor for normal human mammary epithelial cells in vitro (Li et al, 1992; Kenney et al, 1993; Normanno et al, 1994a, b) and its overexpression observed in human malignancies such as breast cancers has been suggested to be involved in tumoral cell growth disregulation (Normanno et al, 1994a, b; Visscher et al, 1997). In order to determine whether AR can act as a mediator of the action of growth factors, we have examined the regulation by EGF of AR-gene expression and -protein secretion, in normal and tumoral breast epithelial cells. Moreover, in an attempt to elucidate further the variety of physiological roles of AR in mammary gland, we investigated whether AR may participate in the regulation of the PA/plasmin system, under both normal and pathological conditions.

Our results demonstrate that EGF affects the expression of AR gene as well as protein secretion, in both normal and tumoral breast epithelial cells. However, whereas a moderate stimulation of AR expression was induced by $10^{-8}$ M EGF in normal mammary cells (210 ± 75% of control), a similar EGF concentration increased AR expression level to 980% and 550% of control in the breast cancer epithelial cell lines MDA-MB231 and MCF-7, respectively. These data are in agreement with reports demonstrating that the expression of some EGF-related growth factors including AR is significantly increased in malignant mammary epithelium relative to normal epithelium (Salomon et al, 1995; Panico et al, 1996). Gene transcription is dependent on the activation of specific transcription factors. In tumoral cells, an increase in amount of oncogenic transcription factors can affect the degree to which they can regulate gene expression (Fry and Farnham, 1999). Thus, in MCF-7 and MDA-MB231 cells, high level of AR mRNA induction by EGF might result from high level of activated transcription factors. Curiously, the inverse pattern was observed regarding the level of secreted protein, since in response to EGF, normal cells were able to release in culture medium 5–10 folds more AR than tumour cells. Martinez-Lacaci et al (1995) have demonstrated that AR was accumulated into the nucleus of MCF-7 cells treated with oestradiol, whereas it was predominantly secreted after treatment with 12-O-tetradecanoyl phorbol-13-acetate. An immunocytochemical examination of MCF-7 and MDA-MB231 cells did not enable the detection of any intracellular accumulation of AR following EGF-treatment (not shown), suggesting that the low level of secreted AR is not due to alterations in secretion process. Alternatively, amphiregulin peptide quantities produced by tumour cells might be influenced by transcriptional or post-transcriptional events such as impaired stability of AR mRNA, and/or by the concomitant accumulation in conditioned medium of proteases that degrade AR. Moreover, various isoforms of AR that differ in the degree of glycosylation and $NH_2$-terminal processing have been described (Shoyab et al, 1988; Johnson et al, 1993b; Martinez-Lacaci et al, 1995). Therefore, the production by tumoral cells of particular AR isoforms undetectable by our enzyme-immunoassay, cannot be excluded and is presently under investigation.

A number of published studies indicate that AR may be involved in a variety of normal physiological events such as differentiation of the mammary gland (Kenney et al, 1995). Nevertheless, AR is more often described for its ability to regulate the proliferation of cultured cells. Thus, AR can function as a potent mitogen on a variety of normal epithelial cells including mammary and prostatic epithelial cells (Li et al, 1992; Kenney et al, 1993). Our results describing an important growth stimulatory activity of AR on normal breast epithelial cells are in agreement with the published data. Also, they are consistent with the demonstration by Kenney et al (1996) that AR can re-establish longitudinal ductal proliferation in quiescent mammary glands of ovariectomized mice suggesting that it may be an important intermediary in glandular development.

Besides these various physiological functions, AR seems to play a special role in tumoral cell progression. To this purpose we have recently demonstrated that antisense expression for AR suppresses tumorigenicity of transformed breast epithelial cells in vivo (Ma et al, 1999). Moreover, the presence of AR in breast cancer cells has been correlated with cancer spread to lymph nodes, suggesting that it might act to enhance tumour metastasis (Lejeune et al, 1993). Plasminogen activation is considered a central process in the regulation of pericellular proteolysis that occurs under both normal and pathological conditions, including cancer invasion. Because high levels of components of the plasminogen activation system, uPA but paradoxically also its inhibitor PAi-1 (Foeckens et al, 1992; Bouchet et al, 1994), have been correlated with a poor prognostic for breast cancers, the role of AR in the regulation of uPA and PAi-1 production by normal and tumoral mammary epithelial cells was further examined. Whereas AR was shown to induce an important stimulation of normal cell proliferation, the levels of both uPA and PAi-1 secreted by these cells were affected neither by AR, nor by EGF. On the contrary, AR induced a dose-dependent accumulation of uPA and PAi-1 in conditioned culture medium of MCF-7 and MDA-MB231 cells but failed, as previously reported by Shoyab et al (1988), to regulate their proliferation. It has to be noted that EGF was less effective than AR in stimulating protease production by tumoral cells. The present study further demonstrated that the increased production of protease induced by AR was accompanied by an augmentation of the number of tumoral cells able to invade reconstituted basement membrane in vitro. The presence of a neutralizing anti-uPA receptor antibody was unable to affect the invasive properties of both untreated and AR-treated MCF-7 cells indicating that uPA is not the principal actor of the degradation of basement membrane by these cells. Given the relative low uPA protein level detected into conditioned medium of MCF-7 cells, we propose that uPA quantity required to degrade matrigel was not attained, even under AR-treatment. Moreover, the ratio PAi-1/uPA appeared clearly in favour of PAi-1, suggesting that uPA activity might be efficiently neutralized. Recently, studies have demonstrated that EGF and AR were able to induce the expression of matrix metalloproteinases (MPPs) in a variety of tumoral cell lines (Kondapaka et al, 1997; Sundareshan et al, 1999). Although these studies have not established a direct link between the increased production of MMPS and cell invasiveness, we suggest that EGF and AR might modulate invasion of MCF-7 cells by up-regulating the expression of member(s) of MPP family. Conversely, the presence of the antibody reversed the invasive effect of AR on MDA-MB231 cells, demonstrating that the growth factor increased the invasiveness of these cells in part by increasing the production of uPA. Moreover, EGF might enhance this process by increasing the production and accumulation of AR protein in the extracellular compartment.

In conclusion, the present study provides additional evidence for the contribution of the AR protein during normal and tumoral mammary development. Firstly, our data reveal differential behaviour of normal *versus* tumoral breast epithelial cells in regard to the action of AR. Thus, while AR is a potent stimulator for normal cell proliferation, it increases the production of uPA and PAi-1 only in tumoral epithelial cells, suggesting that molecular alterations associated to cell transformation may modify the function of AR. AR, as most of EGF-like growth factors, directly interacts with EGF-receptor (ErbB1). The fact that AR elicits distinct biological responses in normal relative to tumoral cells, does not result from differences in EGF-R content, since similar high receptor number were measured in HMECs and MDA-MB231 cells (not shown). However, EGF-like growth factors may also differentially regulate signalling events by activating other ErbB family receptors through a transmodulation mechanism (Riese et al, 1996). A corollary is that the activation of these different receptors results in distinct biological responses. Although the presence of various ErbB family receptors in our HMECs has not been examined, it can be speculated that the differential activity of AR in normal *versus* tumoral cells might result from the activation of different sets of receptors. In a second step, this study demonstrates for the first time that AR might promote invasion and metastasis of a variety of breast tumours by stimulating the PA/plasmin system. Although the physiological relevance of these observations needs to be established in vivo, these data suggest that AR play a special role in the pathogenesis of the breast. Presently, we can assert that the reduction of the tumorigenecity of transformed mammary epithelial cells that results of AR-antisens transfection (Ma et al, 1999) is associated to a reduction of the expression of uPA in tumours developed into *nude* mice (manuscript in preparation). It is probable that a better knowledge of the function of AR in breast tissue might open new perspectives for the diagnosis, prognosis and treatment of breast cancer.

### ACKNOWLEDGEMENTS

The authors wish to thank Jeanne Carbone for excellent technical assistance. We thank Prof Bureau and Dr Bonnier for providing normal breast samples. We also gratefully acknowledge Dr Plowman for providing amphiregulin cDNA probe.

### REFERENCES

Andreasen PA, Kjoller L, Christensen L and Duffy MJ (1997) The urokinase-type plasminogen activator system in cancer metastasis: a review. *Int J Cancer* **72**: 1–22

Bouchet C, Spyratos F, Martin PM, Hacene K, Gentile A and Oglobine J (1994) Prognosis value of urokinase-type plasminogen activator (uPA) and plasminogen activator inhibitors PAI-1 and PAI-2 in breast carcinomas. *Br J Cancer* **69**: 398–405

Ciardiello F, Kim N, Liscia DS, Bianco C, Lidereau R, Merlo G, Callahan R, Brattain M, Greiner J, Szpak C, Kidwell W, Derynck R, Schlom J and Salomon DS (1989) Transforming growth factor α (TGFα) mRNA expression in human breast carcinomas and TGFα activity in the effusions of breast cancer patients. *J Natl Cancer Inst* **81**: 1165–1171

Feinberg AP and Vogelstein BA (1983) Technique for radiolabelling DNA restriction endonuclease fragments to high specific activity. *Anal Biochem* **132**: 6–13

Foeckens JA, Schmitt M, van Putten WL, Peters HA, Bontenbal M, Janicke F and Klijn GJ (1992) Prognostic value of urokinase-type plasminogen activator in 671 primary breast cancer patients. *Cancer Res* **52**: 6101–6105

Fontanini G, De Laurentiis M, Vignati S, Chine S, Lucchi M, Silvestri V, Mussi A, De Placido S, Tortora G, Bianco AR, Gullick W, Angeletti CA, Bevilacqua G and Ciardiello F (1998) Evaluation of epidermal growth factor-related growth factors and receptors and of neoangiogenesis in completely resected stage I-IIIA non-small-cell lung cancer: amphiregulin and microvessel count are independent prognosis indicators of survival. *Clinical Cancer Res* **4**: 241–249

Fry CJ and Farnham PJ (1999) Context-dependent transcriptional regulation. *J Biol Chem* **274**: 29583–29586

Higashiyama S, Abraham JA, Miller J, Fiddes JC and Klagsbrun M (1991) A heparin-binding growth factor secreted by macrophage-like cells that is related to EGF. *Science* **251**: 936–9391

Johnson GR, Saeki T, Gordon A, Shoyab M, Salomon DS and Stromberg K (1992) Autocrine action of amphiregulin in a colon carcinoma cell line and immunocytochemical localization of amphiregulin in human colon. *J Cell Biol* **118**: 741–751

Johnson GR, Kannan B, Shoyab M and Stromberg K (1993a) Amphiregulin induces tyrosine phosphorylation of the epidermal growth factor receptor and $p185^{erbB2}$. Evidence that amphiregulin acts exclusively through the epidermal growth factor receptor at the surface of human epithelial cells. *J Biol Chem* **268**: 2924–2931

Johnson GR, Prigent SA, Gullick WJ and Stromberg K (1993b) Characterization of high and low molecular forms of amphiregulin that differ in glycosylation and peptide core length. *J Biol Chem* **268**: 18835–18843

Kenney N, Johnson G, Selvam MP, Kim N, Cheng-Feng Q, Saeki T, Brandt R, Wallace-Jones B, Ciardiello F, Shoyab M, Plowman G, Day A, Salomon DS and Normanno N (1993) Transforming growth factor α (TGFα) and amphiregulin (AR) as autocrine growth factors in nontransformed, immortalized 184A1N4 human mammary epithelial cells. *Mol Cell Diff* **1**: 163–184

Kenney NJ, Huang RP, Johnson GR, Wu JX, Okamura D, Matheny W, Kordon E, Gullick WJ, Plowman G and Smith GH et al (1995) Detection and location of amphiregulin and Cripto-1 expression in the developing postnatal mouse mammary gland. *Molecular Reproduction and Development* **41**: 277–286

Kenney NJ, Smith GH, Rosenberg K, Cutler ML and Dickson RB (1996) Induction of ductal morphogenesis and lobular hyperplasia by amphiregulin in the mouse mammary gland. *Cell Growth Diff* **7**: 1769–1781

Kondapaka SB, Fridman R and Reddy KB (1997) Epidermal growth factor an amphiregulin up-regulate matrix metalloproteinase-9 (MMP-9) in human breast cancer cells. *Int J Cancer* **70**: 722–726

LeJeune S, Leek R, Horak E, Plowman G, Greenall M and Harris AL (1993) Amphiregulin, epidermal growth factor receptor, and estrogen receptor expression in human primary breast cancer. *Cancer Res* **53**: 3597–3602

Li S, Plowman GD, Buckley SD and Shipley GD (1992) Heparin inhibition of autonomous growth implicates amphiregulin as an autocrine growth factor for normal human mammary epithelial cells. *J Cell Physiol* **153**: 103–111

Ma L, Gauvillé C, Berthois Y, Johnson GR and Calvo F (1999) Antisense expression for amphiregulin suppresses tumorigenicity of a transformed human breast epithelial cell line. *Oncogene* **18**: 6513–6520

Martinez-Lacaci I, Saceda M, Plowman GD, Johnson GR, Normanno N, Salomon DS and Dickson RB (1995) Estrogen and phorbol esters regulate amphiregulin expression by two separate mechanisms in human breast cancer cell lines. *Endocrinology* **136**: 3983–3992

Normanno N, Ciardiello F, Brandt R and Salomon DS (1994a) Epidermal growth factor-related peptides in the pathogenesis of human breast cancer. *Breast Cancer Res Treat* **29**: 11–27

Normanno N, Selvam MP, Qi CF, Saeki T, Johnson G, Kim N, Ciardiello F, Shoyab M, Plowman G, Brandt R, Todaro G and Salomon DS (1994b) Amphiregulin as an autocrine growth factor for c-Ha-ras and c-erbB-2-transformed human mammary epithelial cells. *Proc Natl Acad Sci USA* **91**: 2790–2794

Normanno N, Selvam MP, Bianco C, Damiano V, De Angelis E, Grassi M, Magliulo G, Tortora G, Salomon DS and Ciardiello F (1995) Amphiregulin anti-sense oligodeoxynucleotides inhibit growth and transformation of a human colon carcinoma cell line. *Int J Cancer* **62**: 762–766

Panico L, D'Antonio A, Salvatore G, Mezza E, Tortora G, De Laurentiis M, De Placido S, Giordano T, Merino M, Salomon DS, Gullick WJ, Pettinato G, Schnitt SJ, Bianco AR and Ciardiello F (1996) Differential immunohistochemical detection of transforming growth factor α, amphiregulin and cripto in human normal and malignant breast tissues. *Int J Cancer* **65**: 51–56

Qi CF, Liscia DS, Normanno N, Merlo G, Johnson GR, Gullick WJ, Ciardiello F, Saeki T, Brandt R, Kim N, Kenney N and Salomon DS (1994) Expression of transforming growth factor α, amphiregulin and cripto-1 in human breast carcinomas. *Br J Cancer* **69**: 903–910

 *© 2001 Cancer Research Campaign*

Riese DJ, Kim ED, Elenius K, Buckley S, Klagsbrun M, Plowman GD and Stern DF (1996) The epidermal growth factor receptor couples transforming growth factor-α, heparin-binding epidermal growth factor-like factor, and amphiregulin to Neu, ErbB-3, and ErbB-4. *J Biol Chem* **271**: 20047–20052

Rosenthal EL, Johnson TM, Allen ED, Apel IJ, Punturieri A and Weiss SJ (1998) Role of the plasminogen activator and matrix metalloproteinase systems in epidermal growth factor- and scatter factor-stimulated invasion of carcinoma cells. *Cancer Res* **58**: 5221–5230

Salomon DS, Normanno N, Ciardiello F, Brandt R, Shoyab M and Todaro GJ (1995) The role of amphiregulin in breast cancer. *Breast Cancer Res Treat* **33**: 103–114

Sehgal I, Bailey J, Hitzemann K, Pittelkow MR and Maihle NJ (1994) Epidermal growth factor receptor-dependent stimulation of amphiregulin expression in androgen-stimulated human prostate cancer cells. *Mol Biol Cell* **5**: 339–347

Shoyab M, McDonald VL, Bradley JG and Todaro GJ (1988) Amphiregulin: a bifunctional growth-modulating glycoprotein produced by the phorbol 12-myristate 13-acetate-treated human breast adenocarcinoma cell line MCF-7. *Proc Natl Acad Sci USA* **85**: 6528–6532

Silvy M, Martin PM, Chajry N and Berthois Y (1998) Differential dose-dependent effects of epidermal growth factor on gene expression in A431 cells: evidence for a signal transduction pathway that can bypass Raf-1 activation. *Endocrinology* **139**: 2382–2391

Souttou B, Hamelin R and Crepin M (1994) FGF2 as an autocrine growth factor for immortal human breast epithelial cells. *Cell Growth & Differentiation* **5**: 615–623

Sundareshan P, Nagle RB and Bowden GT (1999) EGF induces the expression of matrilysin in the human prostate adenocarcinoma cell line, LNCaP. *Prostate* **40**: 159–166

Visscher DW, Sarkar FH, Kasunic TC and Reddy KB (1997) Clinicopathological analysis of amphiregulin and heregulin immunostaining in breast neoplasia. *Breast Cancer Res Treat* **45**: 75–80

*© 2001 Cancer Research Campaign*

## References

[PDF_00968] Andreasen P. A., Kjøller L., Christensen L., Duffy M. J. (1997). The urokinase-type plasminogen activator system in cancer metastasis: a review.. Int J Cancer.

[PDF_00971] Bouchet C., Spyratos F., Martin P. M., Hacène K., Gentile A., Oglobine J. (1994). Prognostic value of urokinase-type plasminogen activator (uPA) and plasminogen activator inhibitors PAI-1 and PAI-2 in breast carcinomas.. Br J Cancer.

[PDF_00975] Ciardiello F., Kim N., Liscia D. S., Bianco C., Lidereau R., Merlo G., Callahan R., Greiner J., Szpak C., Kidwell W. (1989). mRNA expression of transforming growth factor alpha in human breast carcinomas and its activity in effusions of breast cancer patients.. J Natl Cancer Inst.

[PDF_00980] Feinberg A. P., Vogelstein B. (1983). A technique for radiolabeling DNA restriction endonuclease fragments to high specific activity.. Anal Biochem.

[PDF_00982] Foekens J. A., Schmitt M., van Putten W. L., Peters H. A., Bontenbal M., Jänicke F., Klijn J. G. (1992). Prognostic value of urokinase-type plasminogen activator in 671 primary breast cancer patients.. Cancer Res.

[PDF_00985] Fontanini G., De Laurentiis M., Vignati S., Chinè S., Lucchi M., Silvestri V., Mussi A., De Placido S., Tortora G., Bianco A. R. (1998). Evaluation of epidermal growth factor-related growth factors and receptors and of neoangiogenesis in completely resected stage I-IIIA non-small-cell lung cancer: amphiregulin and microvessel count are independent prognostic indicators of survival.. Clin Cancer Res.

[PDF_00992] Fry C. J., Farnham P. J. (1999). Context-dependent transcriptional regulation.. J Biol Chem.

[PDF_00994] Higashiyama S., Abraham J. A., Miller J., Fiddes J. C., Klagsbrun M. (1991). A heparin-binding growth factor secreted by macrophage-like cells that is related to EGF.. Science.

[PDF_01001] Johnson G. R., Kannan B., Shoyab M., Stromberg K. (1993). Amphiregulin induces tyrosine phosphorylation of the epidermal growth factor receptor and p185erbB2. Evidence that amphiregulin acts exclusively through the epidermal growth factor receptor at the surface of human epithelial cells.. J Biol Chem.

[PDF_01007] Johnson G. R., Prigent S. A., Gullick W. J., Stromberg K. (1993). Characterization of high and low molecular weight forms of amphiregulin that differ in glycosylation and peptide core length. Evidence that the NH2-terminal region is not critical for bioactivity.. J Biol Chem.

[PDF_00997] Johnson G. R., Saeki T., Gordon A. W., Shoyab M., Salomon D. S., Stromberg K. (1992). Autocrine action of amphiregulin in a colon carcinoma cell line and immunocytochemical localization of amphiregulin in human colon.. J Cell Biol.

[PDF_01016] Kenney N. J., Huang R. P., Johnson G. R., Wu J. X., Okamura D., Matheny W., Kordon E., Gullick W. J., Plowman G., Smith G. H. (1995). Detection and location of amphiregulin and Cripto-1 expression in the developing postnatal mouse mammary gland.. Mol Reprod Dev.

[PDF_01020] Kenney N. J., Smith G. H., Rosenberg K., Cutler M. L., Dickson R. B. (1996). Induction of ductal morphogenesis and lobular hyperplasia by amphiregulin in the mouse mammary gland.. Cell Growth Differ.

[PDF_01023] Kondapaka S. B., Fridman R., Reddy K. B. (1997). Epidermal growth factor and amphiregulin up-regulate matrix metalloproteinase-9 (MMP-9) in human breast cancer cells.. Int J Cancer.

[PDF_01026] LeJeune S., Leek R., Horak E., Plowman G., Greenall M., Harris A. L. (1993). Amphiregulin, epidermal growth factor receptor, and estrogen receptor expression in human primary breast cancer.. Cancer Res.

[PDF_01029] Li S., Plowman G. D., Buckley S. D., Shipley G. D. (1992). Heparin inhibition of autonomous growth implicates amphiregulin as an autocrine growth factor for normal human mammary epithelial cells.. J Cell Physiol.

[PDF_01032] Ma L., Gauvillé C., Berthois Y., Millot G., Johnson G. R., Calvo F. (1999). Antisense expression for amphiregulin suppresses tumorigenicity of a transformed human breast epithelial cell line.. Oncogene.

[PDF_01035] Martínez-Lacaci I., Saceda M., Plowman G. D., Johnson G. R., Normanno N., Salomon D. S., Dickson R. B. (1995). Estrogen and phorbol esters regulate amphiregulin expression by two separate mechanisms in human breast cancer cell lines.. Endocrinology.

[PDF_01039] Normanno N., Ciardiello F., Brandt R., Salomon D. S. (1994). Epidermal growth factor-related peptides in the pathogenesis of human breast cancer.. Breast Cancer Res Treat.

[PDF_01046] Normanno N., Selvam M. P., Bianco C., Damiano V., de Angelis E., Grassi M., Magliulo G., Tortora G., Salomon D. S., Ciardiello F. (1995). Amphiregulin anti-sense oligodeoxynucleotides inhibit growth and transformation of a human colon carcinoma cell line.. Int J Cancer.

[PDF_01042] Normanno N., Selvam M. P., Qi C. F., Saeki T., Johnson G., Kim N., Ciardiello F., Shoyab M., Plowman G., Brandt R. (1994). Amphiregulin as an autocrine growth factor for c-Ha-ras- and c-erbB-2-transformed human mammary epithelial cells.. Proc Natl Acad Sci U S A.

[PDF_01050] Panico L., D'Antonio A., Salvatore G., Mezza E., Tortora G., De Laurentiis M., De Placido S., Giordano T., Merino M., Salomon D. S. (1996). Differential immunohistochemical detection of transforming growth factor alpha, amphiregulin and CRIPTO in human normal and malignant breast tissues.. Int J Cancer.

[PDF_01056] Qi C. F., Liscia D. S., Normanno N., Merlo G., Johnson G. R., Gullick W. J., Ciardiello F., Saeki T., Brandt R., Kim N. (1994). Expression of transforming growth factor alpha, amphiregulin and cripto-1 in human breast carcinomas.. Br J Cancer.

[PDF_01062] Riese D. J., Kim E. D., Elenius K., Buckley S., Klagsbrun M., Plowman G. D., Stern D. F. (1996). The epidermal growth factor receptor couples transforming growth factor-alpha, heparin-binding epidermal growth factor-like factor, and amphiregulin to Neu, ErbB-3, and ErbB-4.. J Biol Chem.

[PDF_01066] Rosenthal E. L., Johnson T. M., Allen E. D., Apel I. J., Punturieri A., Weiss S. J. (1998). Role of the plasminogen activator and matrix metalloproteinase systems in epidermal growth factor- and scatter factor-stimulated invasion of carcinoma cells.. Cancer Res.

[PDF_01070] Salomon D. S., Normanno N., Ciardiello F., Brandt R., Shoyab M., Todaro G. J. (1995). The role of amphiregulin in breast cancer.. Breast Cancer Res Treat.

[PDF_01073] Sehgal I., Bailey J., Hitzemann K., Pittelkow M. R., Maihle N. J. (1994). Epidermal growth factor receptor-dependent stimulation of amphiregulin expression in androgen-stimulated human prostate cancer cells.. Mol Biol Cell.

[PDF_01076] Shoyab M., McDonald V. L., Bradley J. G., Todaro G. J. (1988). Amphiregulin: a bifunctional growth-modulating glycoprotein produced by the phorbol 12-myristate 13-acetate-treated human breast adenocarcinoma cell line MCF-7.. Proc Natl Acad Sci U S A.

[PDF_01080] Silvy M., Martin P. M., Chajry N., Berthois Y. (1998). Differential dose-dependent effects of epidermal growth factor on gene expression in A431 cells: evidence for a signal transduction pathway that can bypass Raf-1 activation.. Endocrinology.

[PDF_01084] Souttou B., Hamelin R., Crépin M. (1994). FGF2 as an autocrine growth factor for immortal human breast epithelial cells.. Cell Growth Differ.

[PDF_01087] Sundareshan P., Nagle R. B., Bowden G. T. (1999). EGF induces the expression of matrilysin in the human prostate adenocarcinoma cell line, LNCaP.. Prostate.

[PDF_01090] Visscher D. W., Sarkar F. H., Kasunic T. C., Reddy K. B. (1997). Clinicopathologic analysis of amphiregulin and heregulin immunostaining in breast neoplasia.. Breast Cancer Res Treat.

